# Association Between Vitamin D Status, Metabolic Syndrome, and Menopausal Type: A Comparative Observational Study in Women with Early and Physiological Menopause

**DOI:** 10.3390/biomedicines14081720

**Published:** 2026-07-31

**Authors:** Anamaria Ardelean, Roxana Furău, Florina Buleu, Nicoleta Mirica, Oana Todut, Ion Petre, Izabella Petre, Tiberiu Buleu, Daian-Ionel Popa, Mircea Iurciuc, Oana Suciu, Cristian George Furău

**Affiliations:** 1 Multidisciplinary Doctoral School, “Vasile Goldis” Western University of Arad, 310414 Arad, Romania; ardelean.anamaria@uvvg.ro (A.A.); todut.oana@uvvg.ro (O.T.); furau.cristian@uvvg.ro (C.G.F.); 2 Department of Life Sciences, “Vasile Goldis” Western University of Arad, 310414 Arad, Romania; 3 Department of General Medicine, Faculty of Medicine, “Vasile Goldis” Western University of Arad, 310414 Arad, Romania; furau.roxana@uvvg.ro; 4 Department of Cardiology, “Victor Babes” University of Medicine and Pharmacy, Eftimie Murgu Square 2, 300041 Timisoara, Romania; 5 County Emergency Clinical Hospital “Pius Brinzeu”, 300732 Timisoara, Romania; petre.izabella@umft.ro (I.P.); tiberiu.buleu@umft.ro (T.B.); 6 Center for Translational Research and Systems Medicine, “Victor Babes” University of Medicine and Pharmacy, 300041 Timisoara, Romania; 7 Department of Kinetotherapy and Motor Skills, Faculty of Physical Education and Sport, West University of Timisoara, 300223 Timisoara, Romania; 8 Department of Functional Sciences, Medical Informatics and Biostatistics Discipline, “Victor Babes” University of Medicine and Pharmacy, 300041 Timisoara, Romania; petre.ion@umft.ro; 9 Department of Obstetrics and Gynecology, “Victor Babes” University of Medicine and Pharmacy, 300041 Timisoara, Romania; 10 Faculty of General Medicine, “Victor Babes” University of Medicine and Pharmacy, 300041 Timisoara, Romania; 11 Research Center for Medical Communication, “Victor Babes” University of Medicine and Pharmacy, 300041 Timisoara, Romania; daian-ionel.popa@umft.ro; 12 Department of Clinic Nursing, “Victor Babes” University of Medicine and Pharmacy, Eftimie Murgu Square 2, 300041 Timisoara, Romania; iurciuc.mircea@umft.ro; 13 Department of Microbiology, “Victor Babes” University of Medicine and Pharmacy, Eftimie Murgu Square 2, 300041 Timisoara, Romania; oana.suciu@umft.ro

**Keywords:** early menopause, physiological menopause, vitamin D deficiency, 25-hydroxyvitamin D, metabolic syndrome, cardiometabolic health, obesity, body mass index, glucose metabolism, women’s health

## Abstract

**Background**: The adverse cardiometabolic outcomes that have been associated with early menopause include obesity, insulin resistance, and metabolic syndrome. Other studies have also linked metabolic dysfunction with vitamin D deficiency. However, the independent association of serum 25-hydroxyvitamin D [25(OH)D] concentrations with metabolic syndrome and type of menopause is unclear. Thus, the present study was designed to assess the association between vitamin D status and metabolic syndrome in women with early menopause compared with those with natural menopause. **Methods**: A total of 301 postmenopausal women were enrolled. Metabolic syndrome was assessed independently according to the NCEP-ATP III and IDF diagnostic definitions. They were classified into two groups: early menopause (EM, n = 79) and physiological menopause (PHYSM, n = 222). Demographic, anthropometric, biochemical, and lifestyle characteristics were recorded. The criteria for diagnosing metabolic syndrome were those of the National Cholesterol Education Program Adult Treatment Panel III (NCEP-ATP III) and the International Diabetes Federation (IDF). Serum 25(OH)D was also categorized as deficiency, insufficiency, or sufficiency. Multivariable logistic regression analyses were used to determine independent predictors of metabolic syndrome. Multivariable linear regression analyses were used to determine the association between serum 25(OH)D and individual metabolic parameters. **Results**: Women with early menopause had a higher body mass index than those with physiological menopause, whereas current smoking was more prevalent among women with physiological menopause. No significant differences were observed between the groups in the prevalence of metabolic syndrome, 25(OH)D concentrations, lipid profile, blood pressure, glycaemic indices, or other biochemical parameters. In multivariable analyses, menopausal status was not independently associated with metabolic syndrome according to either the NCEP-ATP III or IDF criteria. Body mass index was the only independent predictor of metabolic syndrome in the adjusted IDF model (OR 1.2, 95% CI 1.0–1.4; *p* = 0.047). Serum 25(OH)D concentrations were independently associated only with lower fasting glucose levels and were not independently associated with metabolic syndrome or its other components. Although vitamin D categories showed significant associations with metabolic syndrome in unadjusted analyses, these associations were attenuated after adjustment for demographic, anthropometric, menopausal, and lifestyle-related factors. **Conclusions**: Menopausal type was not independently associated with the prevalence of metabolic syndrome or vitamin D status. Although metabolic syndrome prevalence differed across vitamin D categories in unadjusted analyses, these associations were attenuated after adjustment for demographic, anthropometric, menopausal, and lifestyle-related factors. Obesity appeared to be the strongest independent predictor of metabolic syndrome within this cohort. Given the retrospective observational design, these findings should be interpreted cautiously, and prospective longitudinal studies are warranted to confirm these associations.

## 1. Introduction

Menopause is defined as the permanent cessation of menstruation for 12 consecutive months that marks the endpoint of reproductive function in women. It is preceded by a transitional period known as perimenopause or the menopausal transition, during which ovarian follicular activity progressively declines and hormonal fluctuations become increasingly pronounced [1]. Changes in body fat distribution, insulin resistance, lipid metabolism, and vascular function, as well as systemic inflammation, have been identified as important contributors to the increased burden of cardiometabolic disease among aging women during the menopausal transition [2,3,4,5].

Although the average age for natural menopause is between 45 and 55 years, 5–10% of women experience menopause earlier than this. Early menopause is defined as occurring between ages 40 and 45. Recently, early menopause has attracted increasing clinical and scientific attention because prolonged exposure to estrogen deficiency may accelerate metabolic ageing independently of chronological age. Earlier loss of ovarian function may intensify visceral fat redistribution, insulin resistance, endothelial dysfunction, and chronic inflammatory activation, thereby increasing susceptibility to cardiometabolic disease over the life course [6]. Previous studies have associated early menopause with increased risks for cardiovascular disease, osteoporosis, type 2 diabetes mellitus, and premature mortality. However, the biological mechanisms underlying these associations are not completely understood. The degree to which menopausal timing independently contributes to cardiometabolic risk is also controversial [7,8,9].

Metabolic syndrome (MetS) represents one of the most important cardiometabolic disorders affecting postmenopausal women. According to the International Diabetes Federation criteria, metabolic syndrome is defined as the combination of central obesity, elevated blood pressure, impaired glucose regulation, hypertriglyceridemia, and reduced high-density lipoprotein cholesterol concentrations. The relative risk for cardiovascular disease, type 2 diabetes mellitus, and all-cause mortality in individuals with metabolic syndrome was 1.5, 5, and 2, respectively [10]. Recent studies indicate that the prevalence of metabolic syndrome increases significantly after menopause; however, among its components, abdominal obesity, hypertension, and impaired fasting glucose are more common in women who have gone through menopause [11,12]. Furthermore, recent studies have demonstrated that women with premature or early menopause have worse cardiometabolic profiles, and therefore, menopausal timing may affect the development of metabolic dysfunction [2,4,13].

Experimental studies have demonstrated that vitamin D receptors are widely expressed in pancreatic β-cells, vascular smooth muscle, endothelial cells, immune cells [14,15] and adipose tissue, supporting a potential role for vitamin D in insulin secretion, glucose homeostasis, inflammatory regulation, and endothelial function. However, evidence from epidemiological studies remains inconsistent [15,16,17,18]. Vitamin D deficiency is very common among women in perimenopause and postmenopause [19,20]. Several observational studies and meta-analyses have reported a significant association between vitamin D deficiency and increased risk of metabolic syndrome [21,22]. However, a large body of evidence remains conflicting since many of the reported associations are substantially attenuated by adjustment for obesity, physical activity, smoking, and other lifestyle-related factors [16,22]. Consequently, it remains uncertain whether vitamin D deficiency is an independent contributor to cardiometabolic dysfunction or primarily reflects an unfavourable metabolic phenotype [17,22].

The interaction between vitamin D status and menopausal characteristics remains incompletely understood. Recent studies by Muho et al. and Rusu et al. [19,23] have suggested potential links between vitamin D status, age at menopause, and general health at this stage, raising the prospect that vitamin D might be involved in the metabolic and endocrine adjustments taking place during the transition to menopause. Few studies, however, have directly compared vitamin D status and cardiometabolic outcomes between women with premature menopause and those with natural menopause. Little is also known about the possible modifying effect of the type of menopause on the relationship between vitamin D and metabolic syndrome. In our previous study, we showed that vitamin D deficiency was highly prevalent in early and physiological menopause and that serum 25(OH)D concentrations were not consistently related to reproductive hormone levels, which would indicate a limited influence of vitamin D on the endocrine changes in menopause; therefore, further exploration of its association with broader metabolic outcomes was required [24].

Although numerous studies have investigated vitamin D deficiency or metabolic syndrome separately in postmenopausal women, relatively few have simultaneously evaluated menopausal timing, serum 25-hydroxyvitamin D concentrations, and metabolic syndrome using multivariable regression models that account for demographic, anthropometric, and lifestyle-related confounders. Clarifying these relationships may help distinguish independent biological associations from those explained by shared metabolic risk factors.

## 2. Materials and Methods

### 2.1. Study Design and Population

This retrospective observational study was conducted at the Department of Obstetrics and Gynecology, County Clinical Hospital Arad, Romania. Medical records of all eligible postmenopausal women meeting the predefined inclusion and exclusion criteria evaluated between January 2022 and December 2024 were retrospectively reviewed and were enrolled regardless of metabolic syndrome status. Menopause was defined as the absence of menstruation for 12 consecutive months, in accordance with the recommendations of the World Health Organization and the most recent guidelines on the nomenclature of menopause [1,6].

The patients were stratified into two groups according to the age at menopause: Early Menopause (EM), defined as menopause between 40 and 45 years of age, and Physiological Menopause (PHYSM), defined as menopause after 45 years of age [13]. Patients with hormone replacement therapy, vitamin D, or calcium supplementation, any medications known to affect vitamin D metabolism, and women with surgically induced menopause, including those with bilateral oophorectomy or hysterectomy resulting in premature ovarian insufficiency, were excluded from the study. Chronic kidney disease, chronic liver disease, active malignancy, autoimmune disorders, malabsorption syndromes, and incomplete clinical or laboratory data were the additional exclusion criteria.

**Figure 1:**
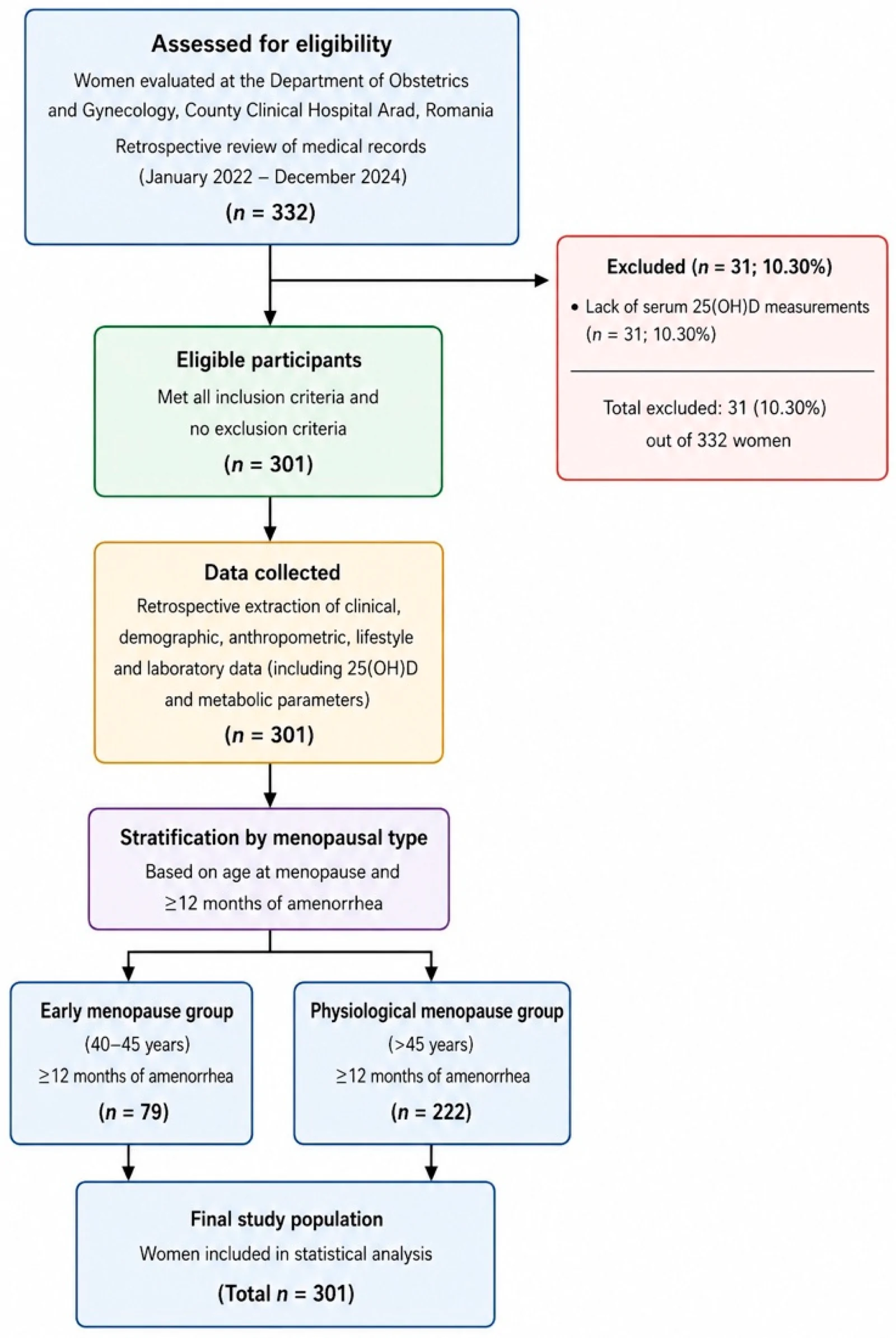
Flowchart of participant selection and classification.

332 women were initially considered eligible to participate in this study. Thirty-one of them (10.3%) were not admitted due to lack of serum 25-hydroxyvitamin D [25(OH)D] results. Thus, the final study population comprised 301 women, with 79 women having early menopause and 222 having physiological menopause. An overview of patient selection and study design is presented in Figure 1.

### 2.2. Clinical and Biochemical Evaluation

All participants underwent a comprehensive clinical and biochemical evaluation. Demographic and lifestyle information such as age, place of residence, level of education, smoking and alcohol habits, physical activity, and age at menopause were collected from the electronic medical records. A clinical examination was performed using standard methods, including measurements of body mass, height, waist circumference, systolic blood pressure (SBP), and diastolic blood pressure (DBP).

Blood pressure measurements were obtained according to current European Society of Cardiology guidelines after a resting period of at least 5 min in the seated position [25]. A calibrated sphygmomanometer (Riester, Jungingen, Germany) equipped with an appropriately sized cuff was used. Measurements were performed on both arms, and the highest value was recorded. Body weight (kg) was measured using a calibrated mechanical scale, while height (m) was assessed using a standardized stadiometer (Fazzini, Vimodrone, Italy). Body mass index (BMI) was calculated as weight in kilograms divided by height in meters squared (kg/m^2^). Waist circumference was measured midway between the lower rib margin and the iliac crest using a non-elastic measuring tape.

Venous blood samples were collected after an overnight fast of 8–12 h. Fasting plasma glucose concentrations were determined using the hexokinase method on a Siemens Dimension RXL-MAX analyzer (Siemens Healthcare Diagnostics, Erlangen, Germany). Serum triglycerides, total cholesterol, high-density lipoprotein cholesterol (HDL-C), and low-density lipoprotein cholesterol (LDL-C) concentrations were measured using standardized enzymatic photometric methods on the same analyzer. Glycated hemoglobin (HbA1c) was assessed by immunoturbidimetric assay standardized according to the Diabetes Control and Complications Trial (DCCT) and certified by the National Glycohemoglobin Standardization Program (NGSP) [26]. Liver function parameters, including aspartate aminotransferase (AST) and alanine aminotransferase (ALT), were determined using routine enzymatic methods.

Serum creatinine concentrations were measured using the Jaffe kinetic method, and the estimated glomerular filtration rate (eGFR) was calculated using the Modification of Diet in Renal Disease (MDRD) equation. Serum 25-hydroxyvitamin D [25(OH)D], the accepted biomarker of vitamin D status, was quantified using the DIAsource 25-OH Vitamin D Total ELISA kit (DIAsource Immunoassays SA, Louvain-la-Neuve, Belgium) according to the manufacturer’s instructions. Vitamin D status was categorized as per current international recommendations into deficiency (≤20 ng/mL), insufficiency (21–30 ng/mL), and sufficiency (>30 ng/mL) [27].

The evaluated metabolic parameters included fasting plasma glucose, triglycerides, HDL-C, LDL-C, total cholesterol, HbA1c, AST, ALT, serum creatinine, eGFR, waist circumference, blood pressure measurements, and serum 25(OH)D concentrations. These variables were subsequently used to assess metabolic syndrome according to both the National Cholesterol Education Program Adult Treatment Panel III (NCEP-ATP III) [28] and International Diabetes Federation (IDF) criteria [29] and to assess the relationship between vitamin D status and cardiometabolic risk factors.

### 2.3. Definition of Metabolic Syndrome

Metabolic syndrome was assessed independently using both the National Cholesterol Education Program Adult Treatment Panel III criteria [28] and the International Diabetes Federation criteria [29]. The two definitions were analysed separately, and participants were classified as either having or not having metabolic syndrome according to each definition. No participant was required to fulfil both definitions simultaneously.

MetS was diagnosed if at least three of the following components were present, according to the NCEP-ATP III definition: abdominal obesity (waist circumference > 88 cm), hypertriglyceridemia (≥150 mg/dL), low high-density lipoprotein cholesterol (HDL-C < 50 mg/dL), elevated blood pressure (≥130/85 mmHg or current antihypertensive treatment), and impaired fasting glucose (≥100 mg/dL) [28].

According to the definition of the IDF, central obesity (waist circumference ≥ 80 cm for European women) was taken as a mandatory criterion, and with this, there should be at least two of the following abnormalities: triglycerides ≥ 150 mg/dL or specific treatment to bring this lipid abnormality, HDL-C < 50 mg/dL or treatment for low HDL-C, blood pressure ≥ 130/85 mmHg or antihypertensive treatment, and fasting plasma glucose ≥100 mg/dL or a prior diagnosis of type 2 diabetes mellitus [29].

### 2.4. Ethical Considerations

This study was carried out in compliance with the ethical principles of the Declaration of Helsinki and its subsequent amendments. The Ethics Committee of the County Clinical Hospital Arad, Romania, granted ethical approval for this study (Approval No. 32/05.06.2024). All the participants signed an informed consent form regarding the use of their clinical and laboratory data, after these were anonymized, for research purposes. Throughout the study, patient confidentiality and anonymity were strictly maintained. All data were analyzed in a de-identified manner.

### 2.5. Statistical Analysis

Statistical analyses were performed using JASP software (Version 0.18.1, JASP Team, Amsterdam, The Netherlands). Continuous variables were assessed for normality using the Shapiro–Wilk test and are presented as mean ± standard deviation (SD) or median with interquartile range (IQR), as appropriate. Categorical variables are reported as frequencies and percentages.

Comparisons between women with early menopause and those with physiological menopause were performed using the independent-samples Student’s *t*-test for normally distributed continuous variables and the Mann–Whitney *U* test for non-normally distributed continuous variables. Comparisons among vitamin D categories were conducted using one-way analysis of variance (ANOVA), followed by Bonferroni-adjusted post hoc comparisons when the overall ANOVA result was statistically significant. Associations between categorical variables were evaluated using Pearson’s chi-square test. Effect sizes were calculated using Cohen’s *d* for comparisons of continuous variables and Cramer’s V for associations between categorical variables.

Pearson’s correlation analysis was performed to examine the relationships between serum 25-hydroxyvitamin D [25(OH)D] concentrations and continuous clinical and biochemical variables.

Multivariable logistic regression analyses were performed to identify factors independently associated with prevalent metabolic syndrome according to the National Cholesterol Education Program Adult Treatment Panel III (NCEP-ATP III) and International Diabetes Federation (IDF) definitions. Covariates were selected a priori based on biological plausibility, previous scientific evidence, and their potential to confound the associations among menopausal status, vitamin D status, and metabolic syndrome. Variable selection was therefore not based solely on statistical significance in univariable analyses.

In the models evaluating the independent association between menopausal status and metabolic syndrome, the predictors included age, body mass index (BMI), menopausal status, residence, educational attainment, smoking status, alcohol intake, and physical activity. Early menopause was used as the reference category for menopausal status. In the models evaluating the independent association between serum 25(OH)D concentrations and metabolic syndrome, the predictors included serum 25(OH)D concentration, age, BMI, menopausal status, residence, educational attainment, smoking status, alcohol intake, and physical activity. Additional logistic regression models included an interaction term between serum 25(OH)D concentration and menopausal status to assess whether menopausal status modified the association between serum 25(OH)D concentrations and metabolic syndrome. Logistic regression results are presented as odds ratios (ORs) with corresponding 95% confidence intervals (CIs) and *p*-values.

Multivariable linear regression analyses were performed to evaluate the independent associations between serum 25(OH)D concentrations and individual cardiometabolic parameters. Each cardiometabolic parameter was entered as the dependent variable in a separate model, with serum 25(OH)D concentration as the principal independent variable. All linear regression models were adjusted for age, BMI, and menopausal status. Results are presented as unstandardized regression coefficients (B), corresponding 95% CIs, standardized regression coefficients (β), and *p*-values.

Before interpretation of the multivariable regression models, multicollinearity was assessed using variance inflation factors (VIFs). VIF values ranged from 1.04 to 2.26 in the logistic regression models and were below 3 in all linear regression models, indicating no evidence of problematic multicollinearity.

The statistical analyses were based on prespecified, hypothesis-driven objectives and evaluated distinct clinical outcomes; therefore, no global adjustment for multiple testing was applied across all analyses. However, Bonferroni-adjusted post hoc comparisons were used following statistically significant omnibus ANOVA results. Findings were interpreted by considering effect estimates, corresponding 95% CIs, and the magnitude and consistency of the observed associations rather than relying solely on statistical significance. Secondary findings were interpreted cautiously in view of the number of comparisons performed.

As this was a retrospective observational study, the sample comprised all participants who met the predefined eligibility criteria during the specified study period. The sample size was therefore determined by the number of eligible medical records available rather than by prospective recruitment, and no a priori sample size calculation was performed.

Numerical results were reported using a level of precision appropriate to the variability of the measurements and the uncertainty of the estimates. Means and corresponding SDs were rounded to the same decimal position. Effect estimates and measures of association were generally reported to two decimal places, with additional precision retained when required to avoid loss of relevant information. *p*-values were generally reported to two decimal places, with additional precision retained for values close to the conventional significance threshold or for very small values. Values below the reporting threshold were expressed using an inequality.

All statistical tests were two-sided, and a *p*-value < 0.05 was considered statistically significant.

## 3. Results

### 3.1. Baseline Characteristics of the Study Population

Table 1 shows the baseline demographic, lifestyle, anthropometric, and biochemical characteristics of the study population. Women with physiological menopause (PHYSM) were significantly older compared to those with early menopause (EM) (50.44 ± 3.04 vs. 42.24 ± 2.48 years, *p* < 0.001). Educational attainment proved different between the groups (*p* = 0.007), as did smoking status (*p* = 0.008) and body mass index (*p* = 0.012). Women with EM had slightly higher BMI compared to women with PHYSM (31.84 ± 5.93 vs. 30.24 ± 4.41 kg/m^2^) while current smoking was more prevalent among women with physiological menopause (18.9% vs. 5.1%). The effect sizes were small and of limited clinical relevance even though statistically significant (Cohen’s d ≤ 0.329; Cramer’s V ≤ 0.181).

No statistically significant between-group differences were observed for place of residence, alcohol intake, physical activity, waist circumference, blood pressure, glycemic parameters, lipid profile, liver enzymes, renal function, serum 25-hydroxyvitamin D [25(OH)D] concentrations, or the prevalence of metabolic syndrome according to either the NCEP-ATP III or IDF definition (Table 1).

### 3.2. Serum 25(OH)D Concentrations According to Menopausal Status

**Figure 2:**
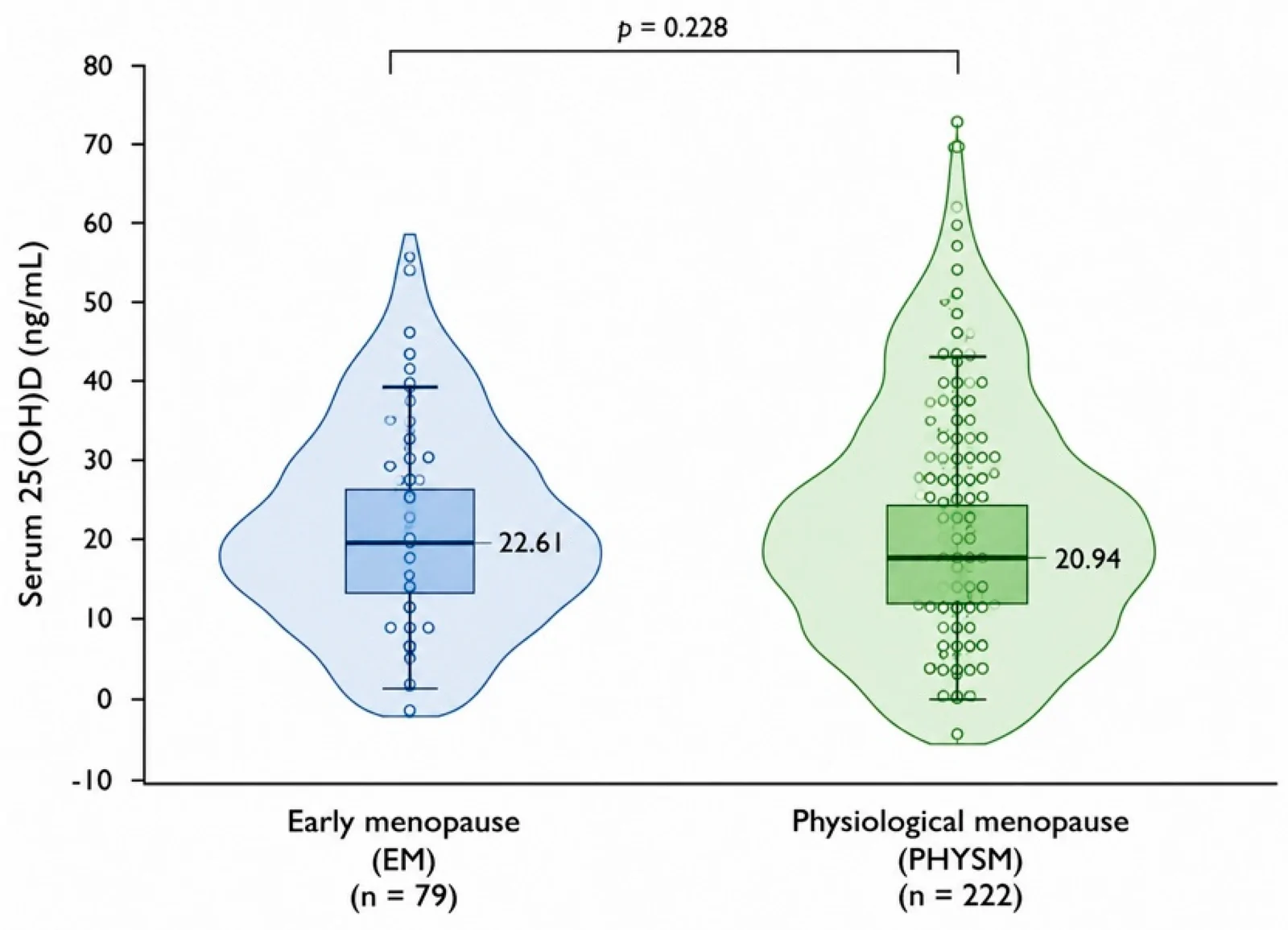
Distribution of serum 25(OH)D concentrations according to menopausal status. The median values are indicated by the central horizontal lines, while boxes represent the interquartile range. No significant between-group difference was observed (*p* = 0.23).

Serum 25(OH)D concentrations did not differ significantly between women with early and physiological menopause (*p* = 0.23). The distributions showed substantial overlap between the two groups, with a wider range of values observed among women with physiological menopause (Figure 2).

### 3.3. Prevalence of Metabolic Syndrome According to Menopausal Status

The prevalence of metabolic syndrome was comparable between women with EM and PHYSM by both diagnostic definitions. According to the NCEP-ATP III criteria, 97.5% of women with EM and 95.0% of women with PHYSM had metabolic syndrome (*p* = 0.36). Similar results were observed with the IDF criteria, with prevalence rates of 97.5% and 95.5%, respectively (*p* = 0.44). These results show that menopausal status did not lead to differences in the prevalence of metabolic syndrome. Table 1 presents detailed prevalence data.

### 3.4. Independent Associations Between Serum 25(OH)D Concentrations and Cardiometabolic Parameters

Multivariable linear regression analyses were performed to evaluate the associations between serum 25-hydroxyvitamin D [25(OH)D] concentrations and individual cardiometabolic parameters, after adjustment for age, body mass index (BMI), and menopausal status (Table 2). Serum 25(OH)D concentrations were not independently associated with waist circumference, triglycerides, HDL cholesterol, systolic blood pressure, diastolic blood pressure, total cholesterol, or LDL cholesterol (all *p* > 0.05).

Higher serum 25(OH)D concentrations were independently associated with lower fasting plasma glucose levels (B = −0.416, 95% CI −0.676 to −0.156, standardized β = −0.180, *p* = 0.002). A small positive association was also observed between serum 25(OH)D concentrations and HbA1c (B = 0.0070, 95% CI 0.0002 to 0.0138, standardized β = 0.119, *p* = 0.043).

### 3.5. Vitamin D Status and Prevalent Metabolic Syndrome

**Figure 3:**
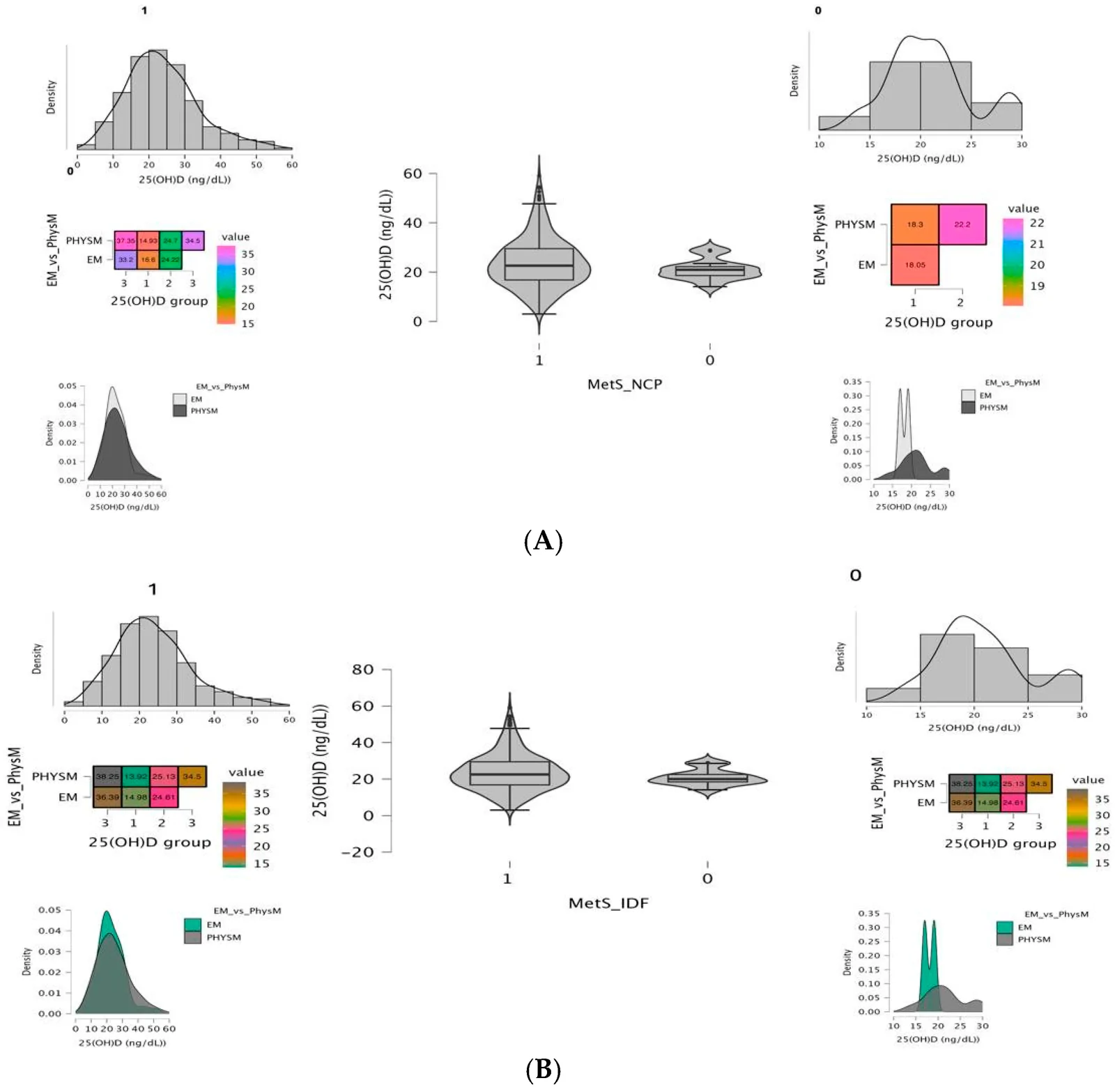
Distribution of serum 25-hydroxyvitamin D [25(OH)D] concentrations according to metabolic syndrome status. (**A**) Metabolic syndrome defined according to the National Cholesterol Education Program Adult Treatment Panel III (NCEP-ATP III) criteria. (**B**) Metabolic syndrome defined according to the International Diabetes Federation (IDF) criteria. Violin-box plots display the distribution of serum 25(OH)D concentrations, with the central horizontal lines representing the medians, the boxes indicating the interquartile ranges, and the dots representing individual participants.

Figure 3 illustrates the distribution of serum 25-hydroxyvitamin D [25(OH)D] concentrations according to metabolic syndrome status. Substantial overlap in serum 25(OH)D distributions was observed between participants with and without metabolic syndrome under both the NCEP-ATP III and IDF definitions. Similar distribution patterns were observed irrespective of the diagnostic criteria applied.

Participants were stratified according to serum 25(OH)D concentrations into vitamin D deficiency (≤20 ng/mL), insufficiency (21–30 ng/mL), and sufficiency (>30 ng/mL). In unadjusted analyses, the prevalence of metabolic syndrome differed significantly across vitamin D categories according to both the NCEP-ATP III criteria (χ^2^ = 18.02, *p* < 0.001, Cramer’s V = 0.245) and the IDF criteria (χ^2^ = 20.95, *p* < 0.001, Cramer’s V = 0.265).

To evaluate the association between serum 25(OH)D concentrations and prevalent metabolic syndrome after adjustment for potential confounders, multivariable logistic regression analyses were adjusted for age, body mass index (BMI), menopausal status, residence, smoking status, alcohol intake, physical activity, and educational attainment. After adjustment, serum 25(OH)D concentrations were not significantly associated with prevalent metabolic syndrome according to either the NCEP-ATP III definition (*p* = 0.235) or the IDF definition (*p* = 0.179). In contrast, BMI remained significantly associated with metabolic syndrome in both adjusted models (NCEP-ATP III: *p* = 0.044; IDF: *p* = 0.032).

Additional interaction analyses were performed to assess whether menopausal status modified the association between serum 25(OH)D concentrations and metabolic syndrome. No statistically significant interaction was observed for either NCEP-ATP III-defined metabolic syndrome (*p* = 0.404) or IDF-defined metabolic syndrome (*p* = 0.434), indicating no evidence that the association between serum 25(OH)D concentrations and metabolic syndrome differed according to menopausal status (Table 3).

Overall, the significant crude associations between vitamin D status and metabolic syndrome prevalence were attenuated after adjustment for anthropometric, demographic, and lifestyle-related factors. Serum 25(OH)D concentrations were therefore not significantly associated with prevalent metabolic syndrome in the adjusted models.

### 3.6. Plasma Glucose Levels

To further investigate the association between serum 25-hydroxyvitamin D [25(OH)D] concentrations and cardiometabolic abnormalities, we evaluated the relationships between serum 25(OH)D concentrations and the individual components of metabolic syndrome.

Correlation analyses revealed a weak inverse correlation between serum 25(OH)D concentrations and fasting plasma glucose levels (r = −0.196, *p* = 0.001). No statistically significant correlations were observed between serum 25(OH)D concentrations and waist circumference (r = 0.045, *p* = 0.440), triglycerides (r = −0.036, *p* = 0.532), HDL cholesterol (r = 0.005, *p* = 0.926), systolic blood pressure (r = 0.017, *p* = 0.766), diastolic blood pressure (r = 0.021, *p* = 0.715), or total cholesterol (r = 0.012, *p* = 0.830).

**Figure 4:**
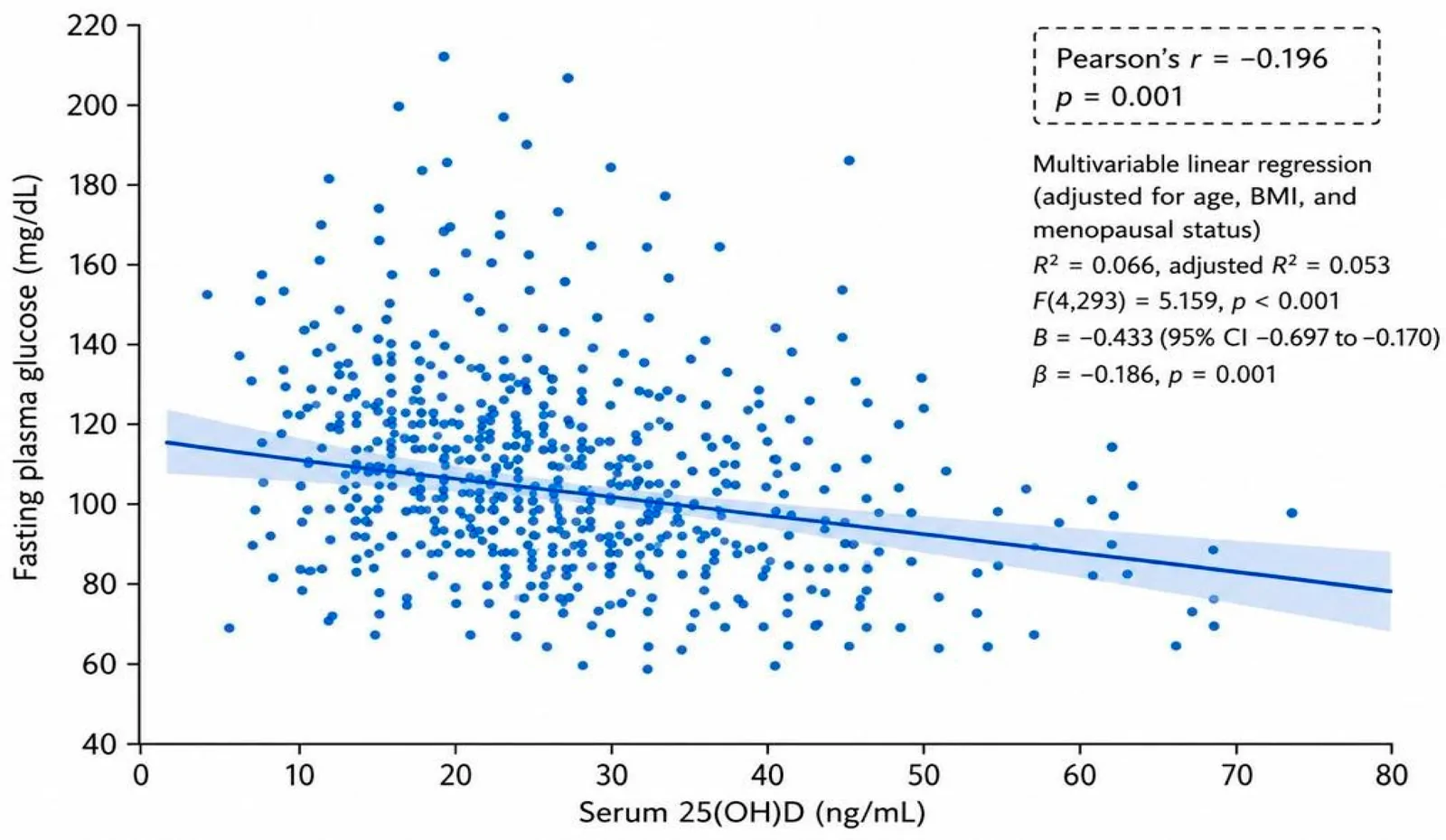
Association between serum 25-hydroxyvitamin D [25(OH)D] concentrations and fasting plasma glucose levels. The scatter plot with a fitted linear regression line and corresponding 95% confidence interval illustrates the inverse relationship between circulating serum 25(OH)D concentrations and fasting plasma glucose levels.

To determine whether the association between serum 25(OH)D concentrations and fasting plasma glucose remained after adjustment for potential confounding factors, a multivariable linear regression analysis was performed with fasting plasma glucose as the dependent variable and serum 25(OH)D concentrations, age, BMI, and menopausal status as independent variables. Higher serum 25(OH)D concentrations remained independently associated with lower fasting plasma glucose levels after adjustment for these covariates (B = −0.416, 95% CI −0.676 to −0.156, standardized β = −0.180, *p* = 0.002). Figure 4 illustrates the inverse relationship between serum 25(OH)D concentrations and fasting plasma glucose levels.

Participants were further stratified according to serum 25(OH)D concentrations into vitamin D deficiency (≤20 ng/mL), insufficiency (21–30 ng/mL), and sufficiency (>30 ng/mL). One-way analysis of variance revealed statistically significant differences among the vitamin D categories in fasting plasma glucose levels (F = 5.13, *p* = 0.006) and diastolic blood pressure (F = 4.00, *p* = 0.019). No statistically significant differences were observed for waist circumference, triglycerides, HDL cholesterol, LDL cholesterol, total cholesterol, HbA1c, or systolic blood pressure (all *p* > 0.05).

Bonferroni-adjusted post hoc analyses showed that fasting plasma glucose levels were significantly higher in women with vitamin D deficiency than in those with vitamin D insufficiency (*p* = 0.025) or vitamin D sufficiency (*p* = 0.019). No statistically significant difference was observed between the vitamin D insufficiency and sufficiency groups (*p* = 1.000).

For diastolic blood pressure, a statistically significant difference was observed between the vitamin D insufficiency and sufficiency groups (*p* = 0.016), with higher values in the vitamin D sufficiency group. Given the absence of a significant linear correlation or an independent association in the adjusted regression model, this isolated post hoc finding should be interpreted cautiously. Detailed ANOVA and post hoc results are presented in Table 4.

Overall, higher serum 25(OH)D concentrations were independently associated with lower fasting plasma glucose levels after adjustment for age, BMI, and menopausal status. No consistent associations were observed between serum 25(OH)D concentrations and the other cardiometabolic parameters. The isolated difference in diastolic blood pressure across vitamin D categories should be interpreted cautiously because it was not supported by the correlation or adjusted regression analyses.

## 4. Discussion

The present study evaluated the relationship between serum 25-hydroxyvitamin D concentrations, metabolic syndrome, and menopausal type in women with early menopause and physiological menopause. Several key observations were made. First, women with early menopause and those with physiological menopause had similar cardiometabolic profiles, including the prevalence of metabolic syndrome, anthropometric measurements, lipid parameters, glycemic indices, and serum 25(OH)D concentrations. Second, menopausal type was not independently related to metabolic syndrome after accounting for potential confounders. Third, although there were significant differences in the prevalence of metabolic syndrome across vitamin D categories in unadjusted analyses, serum 25(OH)D levels were not independently associated with metabolic syndrome after multivariable adjustment. Last, higher serum 25(OH)D levels were independently related to lower fasting plasma glucose and HbA1c levels, indicating a specific relationship between vitamin D status and glucose metabolism.

### 4.1. Metabolic Syndrome in Early and Physiological Menopause

Cardiometabolic parameters and prevalence of metabolic syndrome did not differ significantly between women with early menopause and those with natural menopause in this study. Early menopause has long been believed to signify an elevated risk for cardiovascular and metabolic complications. However, our results indicate that the timing of menopause may not be a major determinant of metabolic syndrome once the menopausal transition is complete. This similarity between the groups may be due to the fact that common risk factors for cardiometabolic health—primarily obesity, age-related metabolic changes, and lifestyle behaviors—contribute more significantly to the manifestations of metabolic syndrome in postmenopausal women.

Our findings further suggest that menopausal timing alone may be insufficient to explain the heterogeneity of cardiometabolic risk observed among postmenopausal women. Han and Choi reported that the association between age at menopause and metabolic syndrome was substantially attenuated after adjusting for socioeconomic and anthropometric variables, which is consistent with the present study’s findings [13]. Similarly, Roa-Díaz et al. emphasized that cardiometabolic risk in postmenopausal women could not be attributed to menopausal timing alone but to various aging, adiposity, hormonal changes, and lifestyle factors [8]. Consequently, the cumulative burden of metabolic risk factors may outweigh the independent contribution of menopausal timing. In addition, reproductive history, including age at menarche, parity, and other reproductive characteristics, has been associated with obesity, hypertension, and long-term cardiovascular risk, suggesting that cardiometabolic health reflects complex interactions between reproductive, genetic, and lifestyle determinants rather than menopausal timing alone.

### 4.2. Vitamin D Status According to Menopausal Type

Serum 25(OH)D concentrations were comparable between women with early menopause and those with physiological menopause, indicating that menopausal timing may not be a major determinant of vitamin D status. Another possible explanation for the absence of differences in serum 25(OH)D concentrations between menopausal groups relates to body composition. Adipose tissue acts as a reservoir for vitamin D, resulting in reduced circulating concentrations among individuals with greater fat mass. Because BMI cannot distinguish between lean and fat mass or quantify visceral adiposity, differences in body composition may partly explain the variability in vitamin D concentrations observed across postmenopausal women. Future studies incorporating dual-energy X-ray absorptiometry, bioelectrical impedance analysis, or imaging-based assessment of body composition may provide a more accurate understanding of these relationships.

Our results are in agreement with the systematic review by Mei et al., which demonstrated a high prevalence of vitamin D insufficiency and deficiency among postmenopausal women worldwide irrespective of menopausal characteristics [20]. Although Muho et al. reported a potential relationship between vitamin D status and menopausal age, the observed associations were modest and likely influenced by multiple confounding factors [23]. In addition, our findings confirm those of our previous study, which reported similar vitamin D concentrations in women with early and physiological menopause, with no obvious relationship between vitamin D status and reproductive hormonal profiles [24]. All in all, the available evidence suggests that vitamin D deficiency is a problem throughout the menopausal years but probably not very much affected by the timing of menopause.

Emerging evidence suggests that the relationship between obesity and vitamin D status is more complex than simple sequestration of this fat-soluble vitamin within adipose tissue. While adipose tissue may reduce circulating serum 25-hydroxyvitamin D concentrations by acting as a storage compartment, additional mechanisms—including volumetric dilution, altered vitamin D metabolism, adipose tissue dysfunction, chronic low-grade inflammation, and obesity-related metabolic disturbances—have also been proposed to explain the lower vitamin D concentrations consistently observed in individuals with obesity. These complementary mechanisms indicate that obesity may influence vitamin D homeostasis through multiple interconnected pathways rather than a single biological process. Consequently, the attenuation of the association between serum 25(OH)D concentrations and metabolic syndrome after adjustment for anthropometric measures in our study likely reflects the complex interaction between adiposity, metabolic dysfunction, and vitamin D physiology rather than an isolated effect of vitamin D deficiency [30].

### 4.3. Vitamin D and Metabolic Syndrome

One of the most important results of this study was the difference between the unadjusted and adjusted analyses regarding the association of vitamin D status with metabolic syndrome. In the unadjusted analysis, lower vitamin D categories were associated with a higher prevalence of metabolic syndrome, but this relationship disappeared in the adjusted analysis that included anthropometric, demographic, and lifestyle-related variables. This pattern of results suggests that vitamin D deficiency is probably only indirectly linked to metabolic syndrome through shared risk factors, rather than through a direct, independent effect.

Obesity is likely to play a central role in this relationship. Vitamin D is a fat-soluble hormone, and as body fat mass increases, its circulating levels decrease because it is stored in adipose tissue. At the same time, obesity is a major driver of insulin resistance, dyslipidemia, chronic inflammation, and other metabolic abnormalities that we commonly refer to as the metabolic syndrome. In this regard, it seems reasonable to consider obesity as a common upstream determinant for both vitamin D deficiency and metabolic syndrome [16,30].

Metabolic syndrome is increasingly recognized as a multifactorial disorder arising from complex interactions among adiposity, insulin resistance, chronic inflammation, hepatic dysfunction, endothelial dysfunction, oxidative stress, and genetic susceptibility [10,31,32,33]. Consequently, attenuation of the association between vitamin D status and metabolic syndrome after multivariable adjustment should not be interpreted as evidence that vitamin D has no biological relevance. Rather, it suggests that circulating 25(OH)D concentrations may reflect the cumulative effects of multiple interrelated metabolic processes that are not fully captured by a single anthropometric measure such as BMI.

The association between obesity and lower circulating serum 25-hydroxyvitamin D [25(OH)D] concentrations is complex and cannot be explained solely by sequestration within adipose tissue. Although vitamin D is a fat-soluble hormone and adipose tissue serves as an important storage site, growing evidence indicates that volumetric dilution represents a major mechanism underlying the lower serum 25(OH)D concentrations observed in individuals with obesity. Drincic et al. proposed that, because vitamin D is distributed throughout a larger body volume in individuals with greater adiposity, circulating concentrations are reduced without necessarily reflecting increased sequestration within fat tissue [34]. These findings are consistent with evidence suggesting that obesity creates a pro-inflammatory milieu that limits the protective effects of vitamin D and promotes chronic metabolic dysfunction [35].

This interpretation is supported by recent reviews and meta-analyses indicating that associations between vitamin D deficiency and metabolic syndrome are substantially weakened after adjustment for obesity and lifestyle-related factors [16,17]. Therefore, low vitamin D concentrations may be better viewed as a marker of adverse metabolic health rather than as an independent determinant of metabolic syndrome development.

### 4.4. Vitamin D and Glucose Metabolism

Although higher serum 25(OH)D concentrations were independently associated with lower fasting glucose concentrations, the observed effect size was relatively modest. Furthermore, the low proportion of explained variance indicates that vitamin D accounts for only a small component of glucose variability. Therefore, these findings should not be interpreted as evidence that vitamin D is a major determinant of glucose metabolism but rather as supportive of a modest association that warrants confirmation in prospective and interventional studies. This hypothesis is supported by several biological mechanisms. Vitamin D receptors are expressed in pancreatic β-cells and peripheral insulin-sensitive tissues. Vitamin D assumes a role in the secretion of insulin, intracellular calcium signaling, and glucose uptake. It may also reduce systemic inflammation and oxidative stress, which have been linked to the development of insulin resistance. All these give biological plausibility to the role of vitamin D in maintaining homeostasis of glucose [14,17,22].

Our findings are consistent with recent studies and meta-analyses that have reported modest but significant associations between vitamin D and glycemic control. In a study by Singh et al., glucose-related parameters improved among postmenopausal women with sufficient vitamin D status; however, recent meta-analyses have shown that vitamin D supplementation benefits fasting glucose and insulin sensitivity in individuals with vitamin D deficiency and increased metabolic risk [16,36]. Glucose metabolism was the only metabolic domain consistently associated with serum 25(OH)D in our cohort; however, lipid parameters, blood pressure, and measures of adiposity did not show independent relationships. This pattern suggests that the metabolic effects of vitamin D, if any, may be more specific to the regulation of glucose than to the wider phenotype of the metabolic syndrome.

### 4.5. Strengths and Limitations

The present study has a few strengths. The participants were assessed for metabolic syndrome using the NCEP-ATP III and IDF definitions, making it possible to check for consistency of findings with different diagnostic criteria. A relatively large cohort of menopausal women was included in the study, which directly compared women with early menopause and physiological menopause. This topic has not been adequately studied in the literature to date. The study also had available detailed demographic, anthropometric, lifestyle, and biochemical data, which allowed for the adjustment of important confounding factors, namely age, BMI, smoking status, alcohol intake, physical activity, educational level, and place of residence. Finally, the study used regression modeling to enable the assessment of independent associations between serum 25(OH)D concentrations, metabolic syndrome, and individual cardiometabolic parameters.

Several limitations should be acknowledged. First, the retrospective cross-sectional design precludes conclusions regarding causality or temporal relationships between vitamin D status and cardiometabolic outcomes. Second, all participants were recruited from a single tertiary care centre, and the study population consisted predominantly of women with metabolic syndrome, limiting the generalisability of the findings and reducing statistical power for comparisons involving participants without metabolic syndrome. Third, information on dietary vitamin D intake, sunlight exposure, seasonal variation in serum 25(OH)D concentrations, and long-term patterns of physical activity was unavailable and therefore could not be included in the analyses. In addition, direct measures of body composition, inflammatory biomarkers, and genetic factors that may influence adiposity, vitamin D metabolism, or cardiometabolic risk were not assessed. Consequently, residual confounding cannot be completely excluded despite adjustment for multiple demographic, anthropometric, menopausal, and lifestyle-related variables. Detailed information on concomitant cardiometabolic medications was not consistently available for all participants due to the retrospective nature of the study and therefore could not be incorporated into the multivariable analyses. Furthermore, because body mass index and waist circumference are surrogate measures of adiposity, they may not fully capture the complex relationship between body fat distribution, vitamin D bioavailability, and metabolic dysfunction.

Despite these limitations, the consistency of the findings across both metabolic syndrome definitions and the multivariable analytical approaches strengthens confidence in the observed associations. Nevertheless, the attenuation of the association between vitamin D status and metabolic syndrome after adjustment suggests that circulating 25(OH)D concentrations may primarily reflect the overall metabolic profile rather than represent an independent determinant of metabolic syndrome. Although higher serum 25(OH)D concentrations were independently associated with lower fasting plasma glucose levels, the observed effect size was modest, and the low proportion of explained variance indicates that vitamin D accounts for only a small component of glucose variability. Therefore, these findings should not be interpreted as evidence that vitamin D is a major determinant of glucose metabolism but rather as supporting a modest independent association that warrants confirmation in well-designed prospective longitudinal studies incorporating direct assessment of body composition, inflammatory biomarkers, and genetic determinants of cardiometabolic risk.

Future analyses should consider years since menopause because cumulative duration of estrogen deficiency may influence cardiometabolic risk independently of chronological age.

## 5. Conclusions

In this study, menopausal type was not independently associated with metabolic syndrome, cardiometabolic risk factors, or serum 25-hydroxyvitamin D (25(OH)D) concentrations. Women with early menopause and physiological menopause had largely similar metabolic and biochemical profiles.

Although lower vitamin D status was associated with a higher prevalence of metabolic syndrome in the unadjusted analyses, this association was attenuated after adjustment for body mass index and other demographic, menopausal, and lifestyle-related confounders. These findings suggest that low serum 25-hydroxyvitamin D [25(OH)D] concentrations may primarily reflect an adverse metabolic profile rather than represent an independent determinant of metabolic syndrome. Higher serum 25(OH)D concentrations remained independently associated with lower fasting plasma glucose levels, whereas the association with HbA1c was modest and should be interpreted cautiously. Overall, these findings support a possible but limited role of vitamin D in glucose homeostasis that warrants further investigation.

Within this cohort, body mass index appeared to be the strongest independent predictor of metabolic syndrome, whereas menopausal timing was not independently associated with metabolic syndrome after adjustment for potential confounders. These findings highlight the importance of comprehensive cardiometabolic risk assessment and weight management in postmenopausal women. Given the observational nature of the study, the observed associations should not be interpreted as causal. Future prospective longitudinal studies incorporating direct measures of body composition, inflammatory biomarkers, and genetic determinants are needed to further clarify the complex relationships among menopausal timing, vitamin D status, adiposity, and cardiometabolic health.

## Figures and Tables

**Table 1:** Baseline characteristics of the study population according to menopausal status.

Variable	EM (n = 79)	PHYSM (n = 222)	Effect Size †	*p*-Value
**Demographics**				
**Age (years)**	42.2 ± 2.5	50.4 ± 3.0	2.82 (d)	<0.001
**Residence**				
**Urban**	44 (55.7%)	119 (53.6%)	0.02 (V)	0.75
**Rural**	35 (44.3%)	103 (46.4%)		
**Educational Attainment**				
**Less than high school**	1 (1.3%)	18 (8.1%)	0.18 (V)	0.007
**High school graduate**	34 (43.0%)	60 (27.0%)		
**More than high school**	44 (55.7%)	144 (64.9%)		
**Alcohol Intake**				
**None**	36 (45.6%)	110 (49.5%)	0.08 (V)	0.55
**Low**	36 (45.6%)	89 (40.1%)		
**Moderate**	6 (7.6%)	14 (6.3%)		
**High**	1 (1.3%)	9 (4.1%)		
**Smoking Status**				
**Never smoker**	44 (55.7%)	93 (41.9%)	0.18 (V)	0.008
**Former smoker**	31 (39.2%)	87 (39.2%)		
**Current smoker**	4 (5.1%)	42 (18.9%)		
**Physical Activity**				
**None**	45 (57.0%)	117 (52.7%)	0.04 (V)	0.77
**1–149 min/week**	29 (36.7%)	87 (39.2%)		
**≥150 min/week**	5 (6.3%)	18 (8.1%)		
**Anthropometric Measurements**				
**Height (cm)**	162 ± 7	162 ± 6	<0.01 (d)	0.99
**Weight (kg)**	77 ± 15	75 ± 13	0.10 (d)	0.45
**BMI (kg/m^2^)**	32 ± 6	30 ± 4	0.33 (d)	0.01
**Metabolic Syndrome Components**				
**Waist circumference (cm)**	103 ± 9	106 ± 10	0.24 (d)	0.07
**Systolic blood pressure (mmHg)**	152 ± 7	153 ± 10	0.10 (d)	0.46
**Diastolic blood pressure** **(mmHg)**	89 ± 7	90 ± 7	0.16 (d)	0.22
**Fasting glucose (mg/dL)**	95 ± 16	100 ± 25	0.19 (d)	0.14
**Triglycerides (mg/dL)**	171 ± 18	172 ± 19	0.03 (d)	0.81
**HDL cholesterol (mg/dL)**	79 ± 21	78 ± 19	0.05 (d)	0.68
**Metabolic syndrome (NCEP-ATP** **III)**	77 (97.5%)	211 (95.0%)	0.05 (V)	0.36
**Metabolic syndrome (IDF)**	77 (97.5%)	212 (95.5%)	0.04 (V)	0.44
**Other Biochemical Parameters**				
**Total cholesterol (mg/dL)**	206 ± 34	206 ± 36	<0.01 (d)	0.99
**LDL cholesterol (mg/dL)**	135 ± 19	139 ± 23	0.17 (d)	0.19
**HbA1c (%)**	5.5 ± 0.6	5.4 ± 0.6	0.04 (d)	0.76
**AST (U/L)**	21 ± 8	23 ± 9	0.18 (d)	0.18
**ALT (U/L)**	19 ± 9	21 ± 11	0.17 (d)	0.19
**25(OH)D (ng/mL)**	22 ± 9	24 ± 10	0.16 (d)	0.23
**Creatinine (mg/dL)**	0.96 ± 0.18	0.97 ± 0.16	0.06 (d)	0.64
**eGFR (mL/min/1.73 m^2^)**	92 ± 6	92 ± 6	0.05 (d)	0.68

† Effect sizes are reported as Cohen’s *d* for continuous variables and Cramer’s *V* for categorical variables. Data are presented as mean ± standard deviation (SD) for continuous variables and n (%) for categorical variables. Continuous variables were compared using independent-samples Student’s *t*-tests, while categorical variables were compared using Pearson’s χ^2^ tests. Statistical significance was defined as *p* < 0.05.

**Table 2:** Multivariable linear regression analyses examining the associations between serum 25-hydroxyvitamin D [25(OH)D] concentrations and cardiometabolic parameters.

Outcome Variable	B (95% CI)	Standardized β	*p*-Value
**Waist circumference (cm)**	0.044 (−0.072 to 0.160)	0.044	0.45
**Triglycerides (mg/dL)**	−0.041 (−0.259 to 0.178)	−0.022	0.71
**HDL cholesterol (mg/dL)**	−0.034 (−0.256 to 0.188)	−0.018	0.76
**Systolic blood pressure (mmHg)**	0.024 (−0.081 to 0.129)	0.026	0.66
**Diastolic blood pressure (mmHg)**	0.030 (−0.048 to 0.108)	0.044	0.45
**Fasting plasma glucose (mg/dL)**	−0.416 (−0.676 to −0.156)	−0.180	0.002 *
**HbA1c (%)**	0.0070 (0.0002 to 0.0138)	0.119	0.043 *
**Total cholesterol (mg/dL)**	0.034 (−0.374 to 0.442)	0.010	0.87
**LDL cholesterol (mg/dL)**	0.062 (−0.185 to 0.310)	0.029	0.62

Data are presented as unstandardized regression coefficients (B) with 95% confidence intervals (CIs), standardized regression coefficients (β), and corresponding *p*-values. Each cardiometabolic parameter was entered as the dependent variable in a separate multivariable linear regression model, with serum 25-hydroxyvitamin D [25(OH)D] concentration as the principal independent variable. All models were adjusted for age, body mass index (BMI), and menopausal status. * Statistically significant at *p* < 0.05.

**Table 3:** Association between serum 25-hydroxyvitamin D [25(OH)D] concentrations and prevalent metabolic syndrome according to NCEP-ATP III and IDF criteria.

Analysis	NCEP-ATP III	IDF
**χ^2^ statistic**	18.02	20.95
**Unadjusted *p*-value**	<0.001	<0.001
**Cramer’s V**	0.245	0.265
**Adjusted association of serum 25(OH)D concentrations**	*p* = 0.235	*p* = 0.179
**Adjusted association of BMI**	*p* = 0.044 *	*p* = 0.032 *
**Interaction term: 25(OH)D × menopausal status**	*p* = 0.404	*p* = 0.434

Data from the unadjusted analyses are presented as Pearson’s χ^2^ statistics, corresponding *p*-values, and Cramer’s V effect sizes. Adjusted associations were evaluated using multivariable logistic regression models including age, BMI, menopausal status, residence, smoking status, alcohol intake, physical activity, and educational attainment. Interaction terms between serum 25(OH)D concentrations and menopausal status were evaluated in separate adjusted models. * Statistically significant at *p* < 0.05.

**Table 4:** Differences in cardiometabolic parameters according to vitamin D status.

Variable	Vitamin D Deficiency, Group 1 (≤20 ng/mL), Mean ± SD	Vitamin D Insufficiency, Group 2 (21–30 ng/mL), Mean ± SD	Vitamin D Sufficiency, Group 3 (>30 ng/mL), Mean ± SD	F Statistic	*p*-Value	Bonferroni-Adjusted Post Hoc Comparison
**Waist circumference (cm)**	105.53 ± 9.56	104.55 ± 10.65	105.48 ± 9.98	0.33	0.72	NS
**Triglycerides (mg/dL)**	171.35 ± 17.88	171.81 ± 22.44	172.49 ± 13.03	0.08	0.93	NS
**HDL cholesterol (mg/dL)**	77.71 ± 18.72	79.36 ± 21.15	78.36 ± 16.97	0.22	0.80	NS
**LDL cholesterol (mg/dL)**	136.64 ± 20.66	138.86 ± 19.43	139.93 ± 26.52	0.57	0.57	NS
**Total cholesterol (mg/dL)**	207.32 ± 34.17	205.40 ± 38.24	206.96 ± 31.68	0.10	0.91	NS
**HbA1c (%)**	5.39 ± 0.53	5.43 ± 0.64	5.54 ± 0.59	1.32	0.27	NS
**Systolic blood pressure (mmHg)**	152.28 ± 9.73	153.12 ± 9.39	153.68 ± 7.25	0.54	0.58	NS
**Fasting plasma glucose (mg/dL)**	103.94 ± 31.16	96.04 ± 16.58	94.18 ± 13.54	5.13	0.006 *	Group 1 vs. Group 2, *p* = 0.025; Group 1 vs. Group 3, *p* = 0.019
**Diastolic blood pressure (mmHg)**	90.06 ± 6.49	88.77 ± 8.02	91.69 ± 4.23	4.00	0.019 *	Group 2 vs. Group 3, *p* = 0.016

Data are presented as mean ± standard deviation (SD). Differences among vitamin D status categories were evaluated using one-way analysis of variance (ANOVA). Bonferroni-adjusted pairwise comparisons were performed when the overall ANOVA result was statistically significant. Group 1: vitamin D deficiency (≤20 ng/mL); Group 2: vitamin D insufficiency (21–30 ng/mL); Group 3: vitamin D sufficiency (>30 ng/mL). * Statistically significant at *p* < 0.05.

## Data Availability

The original contributions presented in this study are included in the article. Further inquiries can be directed to the corresponding authors.

## Abbreviations

Abbreviation	Definition
25(OH)D	25-Hydroxyvitamin D
ALT	Alanine aminotransferase
ANOVA	Analysis of variance
AST	Aspartate aminotransferase
BMI	Body mass index
CI	Confidence interval
DBP	Diastolic blood pressure
DCCT	Diabetes Control and Complications Trial
ELISA	Enzyme-linked immunosorbent assay
EM	Early menopause
eGFR	Estimated glomerular filtration rate
HbA1c	Glycated hemoglobin
HDL-C	High-density lipoprotein cholesterol
IDF	International Diabetes Federation
IQR	Interquartile range
LDL-C	Low-density lipoprotein cholesterol
MDRD	Modification of Diet in Renal Disease
MetS	Metabolic syndrome
NCEP-ATP III	National Cholesterol Education Program Adult Treatment Panel III
NGSP	National Glycohemoglobin Standardization Program
NS	Not statistically significant
OR	Odds ratio
PHYSM	Physiological menopause
SBP	Systolic blood pressure
SD	Standard deviation
SPSS	Statistical Package for the Social Sciences
VIF	Variance inflation factor
WHO	World Health Organization
